# Smartphone apps for managing alcohol consumption: a literature review

**DOI:** 10.1186/s13722-020-00190-x

**Published:** 2020-05-07

**Authors:** Stephanie Colbert, Louise Thornton, Robyn Richmond

**Affiliations:** 1 School Public Health and Community Medicine, University of New South Wales, Kensington, Australia; 2 The Matilda Centre for Research in Mental Health and Substance Use, University of Sydney, Sydney, Australia

**Keywords:** Smartphone, Mobile phone, Apps, Application, Alcohol, Drinking

## Abstract

**Background:**

Smartphone applications (apps) designed to assist users to reduce hazardous and harmful alcohol consumption show potential as an inexpensive alternative to traditional brief intervention in primary care. The aim of this paper is to provide an overview of the literature on alcohol reduction apps and the availability of evidenced-based apps on top commercial app stores.

**Methods:**

We reviewed literature through to December 2019 using the databases PubMed, MEDLINE, PsycINFO and Google Scholar and keyword search terms smartphone/mobile/phone AND application/app AND alcohol. Articles were included if the primary intervention was a smartphone app and the study measured participant changes in frequency or volume of alcohol consumption.

**Results:**

21 relevant articles were identified that evaluated 19 unique smartphone apps. Of the 19 unique apps, seven were designed for use among youth and 12 in adult populations. The available evidence for the efficacy of alcohol reduction apps among youth is inconclusive, with results from these evaluations not showing a clear benefit in reducing alcohol consumption compared to control groups. The results of apps designed for adult populations appears more promising, but results are still mixed. Of the 19 alcohol reduction apps that have been evaluated only eight of these are currently publicly available in commercial app stores. Of these eight apps, only four were demonstrated in the literature to assist with reducing alcohol consumption.

**Conclusion:**

The evidence for alcohol reduction apps is promising but inconclusive. Few apps that have been evaluated in the scientific literature are currently available for download in commercial app stores.

## Introduction

Alcohol use is a leading risk factor for death and disability, accounting for 5.1% of the global burden of disease [1]. Alcohol use has been linked to over 200 health conditions, including cardiovascular disease, cancers, dementia, road injuries, violence and suicides [1]. Despite the risk of adverse consequences, many people still consume alcohol at levels that increase their risk of harm [1–3].

Face-to-face brief interventions delivered in primary care appear to be effective in reducing alcohol consumption in patients drinking at hazardous and harmful levels [4]. A 2018 Cochrane review found that hazardous or harmful drinkers who received brief intervention consumed a mean of 20 grams per week less alcohol (95% CI = 28 to 12) after 1 year than participants who received minimal or no intervention [4]. However, several barriers exist which limit the reach of brief interventions in primary care, including heavy clinician workload, a lack of appropriate training and limited time in consultations to manage competing health priorities [5]. For patients, barriers for not seeking treatment include the stigma associated with substance use disorders, and logistical and financial barriers [6].

The use of digital interventions (e.g. websites and smartphone applications), has the potential to overcome some of the barriers by offering an inexpensive, accessible method to deliver education, support and monitoring through personal devices. There have been several meta-analyses conducted evaluating the effectiveness of digital interventions for reducing alcohol consumption that have consistently found small reductions in the intervention groups compared to controls [7–9]. A 2017 Cochrane review reported that participants using a digital intervention drank approximately 23 grams of alcohol per week less than controls who received information only or usual care [10].

In more recent years the focus in digital health has shifted to smartphone apps, with more than 318,000 health apps currently available on top app stores and an estimated 200 new apps being added daily [11]. Smartphone apps have several advantages over standard websites, including the ability to be used without internet access and the ability to provide a more personalised experience for users with options for customization. With near ubiquitous smartphone ownership in countries such as the United States [12] and Australia [13], if effective, these apps have great potential to improve health outcomes.

There are a large number of apps currently available in commercial app stores that are aimed at assisting users to reduce their alcohol intake, however few have been evaluated. The aim of this paper is to provide an overview of the literature on alcohol reduction apps and the availability of evidenced-based apps on top commercial app stores.

## Method

We conducted a literature review of English language articles published until 4 December 2019 which evaluated the efficacy or effectiveness of a smartphone app in reducing alcohol consumption. The databases searched were PubMed, MEDLINE, PsycINFO and Google Scholar. Keyword search terms were smartphone/mobile/phone AND application/app AND alcohol. Titles and abstracts of all potentially relevant articles were reviewed for possible inclusion by one author (SC). Articles were included if the primary intervention was a smartphone app (native app not a web-based app) and the study measured participant changes in frequency or volume of alcohol consumption. Reference lists from each article were also examined to identify articles that may have been missed in the initial search.

For each unique app identified in the papers we searched for its availability in the top two app stores, Google Play and iTunes in January–February 2020. We searched the United States (US), Canadian, United Kingdom (UK), Irish, Australian and New Zealand (NZ) iTunes and Google Play stores for all apps, plus the iTunes and Google Play stores of the country where the app was developed, e.g. if the app was developed in Sweden we also searched the Swedish iTunes and Google Play stores. If the app could not be found in any Google Play or iTunes stores the corresponding author of the study was emailed to enquire about the app’s availability.

## Results

The database searches returned 518 results of which 21 were studies evaluating whether use of a smartphone app reduced participants’ frequency or volume of alcohol consumption [14–34]. These 21 articles evaluated 19 unique smartphone apps. Eight of these apps could be found in the iTunes and/or Google Play stores of at least one of the following countries: US, Canada, UK, Ireland, Australia and NZ [15, 19, 20, 22–24, 26, 30, 34]. For the 11 apps that could not be found, corresponding authors of the papers were emailed and three replied: one confirming the app is no longer available in app stores [14, 21], one advising the app had been revised and was available under another name [24], and one advising that the app had been temporarily taken down to be updated [17].

Of the 19 unique apps, seven were designed for use among youth [14–21] and 12 in adult populations [22–34]. The results of these studies are summarised in Table 1.

**Table 1:** Alcohol reduction apps evaluated in the peer reviewed literature

App name	Target group	Research methods	Results
Alcooquizz [22]	Individuals in the general population with unhealthy alcohol use	Study design: Pilot RCT Participants: 977 participants with unhealthy alcohol use recruited online Intervention group: 416 participants offered the Alcooquizz app for download. Alcoquizz app contains five active components, including providing personalised feedback, a self-monitoring tool, a designated driver tool, eBAC calculator, and fact sheets on alcohol use and its consequences Control group: 516 participants not offered any intervention Follow-up: 6 months	No significant difference in reported SD consumed/week between intervention and control group at 6-months–IRR 0.93 (95% CI 0.84, 1.03) In a per protocol analysis where participants in the intervention group who did not actually download the app were excluded the difference in SD/week at 6 months was significant – IRR 0.88 (95%CI = 0.78, 0.99) Availability: Free to download in Australian, NZ, US, UK, Irish, Canadian and Swiss iTunes stores. Unable to find app in any Google Play stores
Drink less [23, 34]	Individuals in the general population who drink excessively	Study design: 2^5^ Factorial RCT to evaluate the five intervention components (modules) of the app Participants: Original trial of 672 participants living in the UK who downloaded the app in a commercial app store and reported drinking excessively [23]. Subsequently, extended recruitment of an additional 1914 participants occurred and all the data were analysed [34]. Total sample size was 2586 but only 342 (13.2%) completed follow-up. Intervention: The Drink Less app is centered around alcohol reduction goal setting with five independent intervention modules: normative feedback, cognitive bias re-training, self-monitoring and feedback, action planning, and identity change Participants were randomly assigned different versions of the app with different combinations of either “enhanced” or “minimal functionality” of the five modules (active components were removed in the minimal functionality versions, which acted as the control) Follow-up: 4-weeks	Results indicated that there are no large main effects of the enhanced version of individual components on SD/week (0.22 < BF < 0.83) or AUDIT score (0.14 < BF < 0.98) compared to minimal versions In an additional exploratory analysis, participants receiving four of the enhanced components averaged a greater reduction in SD/week than those not receiving any (21.6 versus 12.1 SDs), but the data were insensitive (BF = 1.42) Availability: Only available for download in UK iTunes store (free). Unable to find app in iTunes stores in other countries, or in any Google Play stores
Drinkaware [26]	Individuals in the general population who want to reduce their alcohol consumption	Study design: Mixed-methods sequential explanatory design Intervention: Drinkaware app has four main features: calculate units of alcohol and energy in drinks and track consumption; information about the impacts of drinking on health; set goals; define geographical locations that may be triggers where support may be required Quantitative: Usage data from 119,713 individuals who downloaded the app over a 13-month period analysed. Additional analysis performed on 12-weeks of usage data of a sub-set of 3401 (2.8%) ‘engaged users’ Qualitative: of the 119,713 users 3491 who had supplied contact details within the app were sent a feedback survey. 189 of these users completed the survey, and of these 21 were recruited for in-depth interview.	*Quantitative findings:* ‘Engaged users’ self-reported a reduction in alcohol consumption over time, but almost all of the change occurred in the first week after downloading the app (reduction of 4.9 units after 1 week, P < 0.05) then consumption plateaued *Qualitative findings:* Mixed feedback received regarding each individual app feature, although there was a common preference for more personalised content Availability: Only available for download in UK iTunes store (free). Unable to find app in iTunes stores in other countries, or in any Google Play stores
Daybreak [30]	Individuals in the general population drinking at risky levels	Study design: Originally designed as RCT but due to technical errors there was no control group Participants: 793 participants in the general population who accessed the Daybreak app and reported AUDIT scores > 7. Half were originally intended to also receive online coaching via real-time chat messages, with the non-coaching group acting as controls. However, due to a technical error some controls could also access the coaching so the main analysis comprised all participants Intervention: Daybreak program aims to assist users to change their relationship with alcohol with four main features: weekly check-ins, peer support, behavioural experiments and health coaching Follow-up: 1 month and 3 months	Reductions in AUDIT-C scores from a mean of 9.1 to 5.8 (P < 0.001) at 3-months follow-up Accessing online coaching was not associated with improved outcomes. Availability: Available free on the Australian iTunes and Google Play stores. 21-day free trial available in iTunes and Google Play stores from other countries, and at a cost after the trial period
Self-Record (Jibun Kiroku) [31]	Full-time workers experiencing mild to moderate psychological distress	Study design: Non-randomised pilot study with control group Participants: 557 Japanese full-time workers aged 20-59 years who were experiencing mild to moderate psychological distress (Kessler Psychological Distress Scale score between 5 and 12), who indicated an interest in self-monitoring smartphone apps. Recruited by a research and marketing company Intervention group: 306 participants who self-selected to the intervention group by agreeing to use to the Self Record app. The Self Record app was designed primarily to improve psychological distress, not to reduce alcohol consumption. However, the authors expected that a reduction in psychological distress would lead to reduction in alcohol consumption so tested this, in addition to measuring changes in psychological distress. The app focuses on self-monitoring and awareness of negative thoughts, daily activities, and daily mood, and provides psychoeducation Control group: 251 participants who did not agree to use the Self Record app. Given only baseline and follow-up surveys, nothing else Follow-up: 4 weeks	*Intention*-*to*-*treat analysis alcohol outcomes:* Participants in the intervention group reported increased typical drinking (η^2^ = 0.009) and heavy drinking (η^2^ = 0.001) compared to controls over the study period. The effect size was small but significant *Per*-*protocol analysis alcohol outcomes:* After excluding participants who discontinued using the app after the first day (65% of intervention group) and those who incorrectly answered a validity check item (65% of all participants), results still showed that the intervention group reported significantly increased typical drinking (η^2^ = 0.005), and heavy drinking (η^2^ = 0.007) compared to those in the control group Availability: Unable to find app in any iTunes or Google Play stores
Cue exposure therapy (CET) [32]	Designed as aftercare for individuals who have recently completed an outpatient alcohol treatment program	Study design: Investigator-blinded parallel RCT Participants: 164 participants were recruited at the end of a 3-month primary treatment program for AUD at an outpatient alcohol treatment clinic in Denmark. Aimed to compare aftercare treatment approaches CET therapy group: 54 participants randomised to receive group CET therapy with urge-specific coping skills (USCS), conducted by a therapist for 4 × 2 h sessions over 8 weeks. CET with USCS aims to teach coping skills to reduce cravings, which individuals the practise when exposed to cues to induce cravings CET app group: 54 participants randomised to receive then CET app, which is based on the same treatment principles as the CET with USCS therapy, transformed into a fully automated mobile phone app. The app contained alcohol exposure videos which imitated sessions with a therapist Control group: 56 participants received aftercare as usual, which included 1 × 1 h individual session 8 weeks after the completion of the treatment program Follow-up: 2 and 6 months	No significant differences were found between the intervention groups (CET therapy group and CET app group) compared with the control group on alcohol consumption outcomes over time Similarly, no differences were found when comparing CET therapy and CET app groups Therefore, CET with USCS delivered as aftercare either via group sessions or a mobile phone app did not increase the effectiveness of usual aftercare for individuals with AUD Availability: Unable to find app in any iTunes or Google Play stores
SIDEAL [27] Acronym of Spanish words = Innovative Support to the Alcohol Dependent Patient	Individuals with alcohol dependence being treated in an outpatient setting	Study design: Pilot study with no control group. Aimed to assess the app’s usability and efficacy at reducing alcohol consumption Participants: 24 participants being treated for alcohol dependence as outpatients Intervention: All participants received the SIDEAL app plus treatment as usual. The SIDEAL app was developed with MI as its theoretical foundation and has six main functionalities: consumption register; pharmacological adherence register; calendar and reminder system for treatment appointments; medical information and psychoeducation on alcohol dependence; questionnaires (addressing cravings, progress etc.); feedback Follow-up: 6-week trial	Reported BD days reduced from 25.5 days in the 6-weeks prior to 5.8 days during the 6-week study period (P < 0.001). SD consumed/day also reduced from 6.5 to 1.9 SD/day (P < 0.001) Availability: Unable to find app in any iTunes or Google Play stores
A-CHESS Addiction– Comprehensive Health Enhancement Support System [25]	Designed to improve continuing care in adults with alcohol use disorders	Study design: Unmasked RCT Participants: 349 participants leaving residential care who met criteria for DSM-IV alcohol dependence when they entered residential treatment Intervention group: 170 participants given treatment as usual plus a smartphone with the A-CHESS app installed for 8-months, followed by a 4-month period with treatment as usual only. The A-CHESS app has its theoretical basis in self-determination theory, aiming to improve its users’ competence, social relatedness, and motivation Control group: 179 participants leaving residential care given treatment as usual only for 12 months Follow-up: 4, 8 and 12 months	Across the 3 follow-up periods there were significantly fewer risky drinking days (days when > 4 SD for men and > 3 SD for women consumed in under 2 h) on average reported in the intervention group compared to control (1.39 vs 2.75 days/month, P = 0.003) Participants in the intervention group were also more likely than controls to report abstinence at all 3 follow-up points (P = 0.03) Availability: Available to download in Australian, NZ, US, UK, Irish, Canadian and Swiss iTunes stores but a provider code/login is needed. Unable to find app in any Google Play stores
CASA-CHESS (A-CHESS translated and adapted for Latinx Spanish-speakers) [33]	Designed to improve continuing care in adults with alcohol use disorders.	Study design: Single group pre-post study. No control group Participants: 79 Spanish-speaking participants leaving a residential alcohol and other drug treatment program in the north eastern USA. Authors separated participants into two subgroups for the analysis based on how long they used the CASA-CHESS app for – > 4 months (n = 58) or < 4 months (n = 21) – and compared the outcomes in their analysis Intervention: All participants were given treatment as usual plus an Android smartphone equipped with the CASA-CHESS app installed for 8-months. The CASA-CHESS app was developed by adapting A-CHESS (described above) to be linguistically and culturally relevant for Latinx Spanish-speaking adults in recovery for alcohol or other drug use disorders Follow-up: Baseline data (leaving residential care) was compared to 6 month follow-up	*Alcohol*-*related outcomes:* Alcohol use did not differ significantly between baseline and 6 months for the overall sample (P = 0.219), or between baseline and 6 months for either of the subgroups (> 4 months, P = 0.625 and < 4 months P = -). Logistic regression analyses examining between subject effects for the > 4 months and < 4 months subgroups also found no significant difference in alcohol use between the groups Availability: Unable to find app in any iTunes or Google Play stores
SoberDiary [28]	Individuals recovering from alcohol dependence undergoing outpatient treatment	Study design: Hybrid effectiveness-implementation design Participants: 38 participants undergoing an outpatient program for alcohol dependence Intervention: All participants given the SoberDiary system, which consists of an app coupled to a bluetooth breathalyser that measures the BrAC. The app contains four functional modules: alcohol use detection; feedback on progress; a self-fulfilment guide; and skills enhancement (managing cravings, high-risk situations etc.) 19 participants were classified as highly adherent (HA) and 19 as less adherent (LA), according their total usage of SoberDiary system throughout the study period. These groups were compared Follow-up: Followed-up at 1, 2, 4, 8, and 12 weeks	Better compliance in the using the app was associated with better outcomes in *some* of the measured drinking outcomes HA participants recorded significantly fewer drinking days/week and SD consumed/week, a higher cumulative number of abstinence days and a higher abstinence rate compared to the LA group. No significant differences observed in the number of heavy drinking days/week, number of drinks per drinking day or the time to relapse Availability: Unable to find app in any iTunes or Google Play stores
LBMI-A (Location-Based Monitoring and Intervention for Alcohol Use Disorders) [24]	Developed for use in people with alcohol use disorder	Study design: Pilot study (not randomised) with intervention and control group Participants: 60 participants who met DSM-V criteria for an alcohol use disorder and were motivated to change their drinking recruited using flyers, radio and newspaper advertisements Intervention group: 31 participants were given the LBMI-A app. The app consists of seven psychoeducation modules each with corresponding practical tools, including modules aimed at managing cravings, providing feedback, and improving problem-solving and drink refusal skills Comparison group: 29 participants given a single Internet-based brief motivational intervention, plus an information booklet on the health consequences of alcohol consumption Follow-up: 6-weeks	Only the LBMI-A group experienced a statistically significant increase in percent days abstinent over the 6-weeks (P < 0.001) the comparison group did not (P = 0.324) Both groups reported statistically significant decreases in SD/week and heavy drinking days at 6-weeks compared to baseline figures Availability: The original LBMI-A app is not available in any iTunes or Google Play stores, however, a revised version of the app called Step Away can be accessed through the Australian, NZ, US, UK, Irish and Canadian iTunes stores. Unable to find Step Away app in Google Play stores
HealthCall-S [29]	Developed for use by alcohol dependent individuals living with HIV	Study design: RCT pilot study Participants: 47 participants living with HIV who had used non-injecting drugs ≥ 4 days and binge drank at least once in the previous 30 days, recruited via ads in newspapers and pamphlets in waiting rooms Intervention group: 23 participants given MI + HealthCall-S app at baseline, then brief MI booster session at 30 and 60 days. The HealthCall-S app is an adaption of a previously trialled interactive voice response resource (HealthCall-IVR). HealthCall is based on 3 main aspects: self-monitoring, positive reinforcement and personalised feedback Control group: 24 participants given MI at baseline and brief booster session at 30 and 60 days Follow-up: 30 days and 60 days	*Alcohol*-*related outcomes:* Reductions in the number of drinking days and mean SD/day in the previous month were greater in the intervention group compared to controls at 60-days follow-up but this difference was not significant (P = 0.09 and P = 0.11 respectively) Other drug-related outcomes did show significantly greater reductions in the intervention group compared to controls Availability: Unable to find app in any iTunes or Google Play stores
Ray’s Night Out [15]	Designed to increase alcohol knowledge and reduce alcohol use in young people	Study design: RCT comparing immediate versus 1-month delayed-access to app Participants: 197 participants aged 16 to 25 years who drank alcohol in the previous month recruited through university student emails and youth relevant websites Intervention group: 101 participants given immediate access to the Ray’s Night Out app, which centres around a red panda avatar called Ray who users take on a virtual night out and are provided with information about the consequences of drinking and strategies for managing alcohol consumption. The aim is that users will gain knowledge, motivation and skills and reduce their alcohol consumption Control group: 96 participants given delayed-access to the Ray’s Night Out app after 1 month Follow-up: 1, 2, 3 and 6 months	No significant differences in alcohol use measures or related harms were found Participants in the immediate access group achieved an increase in alcohol knowledge (measured using 16-item questionnaire, scores ranging 0 to 16) at 1-month follow-up compared to baseline (mean difference = 0.90, 95% CI: 0.49, 1.30) but the delayed-access group did not (mean difference = 0.32, 95% CI: −0.07, 0.72) Availability: Free to download in Australian, NZ, US, UK, Irish and Canadian iTunes and Google Play stores
Promillekoll and Party Planner [14, 21]	Aims to promote safe drinking choices on party occasions among university students	Study design: RCT with two intervention groups compared to a control group Participants: 2166 Swedish university students with hazardous drinking (AUDIT score ≥ 6 for women and ≥ 8 for men), recruited via email and Facebook followed-up for 7 weeks Promillekoll iPhone/Android app group: 722 participants given the Promillekoll app, which contains a real-time BAC calculator and offers a number of strategies to maintain alcohol consumption below harmful levels Party Planner web-based app group: 722 participants given the Party Planner app, which is similar to Promillekoll app with additional functionality of simulating or planning a drinking event beforehand and then comparing the simulation to the real-time event afterwards Control group: 722 participants received no intervention or information between study registration and follow-up Follow-up: 7-weeks. Data for a subgroup of participants with hazardous but not excessive drinking levels (n = 1157, baseline weekly drinking > 9 SD/week for women and > 14 SD/week for men) followed up at 7, 14 and 20 weeks	*7*-*week follow*-*up, all participants:* The only significant difference between either group and controls was that the Promillekoll group showed a higher relative risk over 7 weeks for excessive drinking in comparison to controls (OR 1.83; P <=0.01; CI = 1.02; 2.99) *7, 14, and 20*-*week follow*-*up for non*-*excessive drinkers:* Estimated average percentage BAC levels for the week were significantly lower for both the PartyPlanner and Promillekoll groups compared to controls at 7, 14, and 20 weeks (P = 0.001 at 7,14,20-weeks for each group compared to controls). Binge drinking occasions/week were lower for the Promillekoll group compared to controls at 7-weeks (P = 0.04), 14-weeks (P = 0.022) and 20-weeks (P = 0.043), but only at 14 weeks for the PartyPlanner group compared to controls (P = 0.046) Availability: The Promillekoll is not available in any iTunes or Google Play stores. The PartyPlanner web-based app is also no longer available on the web
TeleCoach [16]	Young people who drink excessively	Study design: Randomised trial with two intervention groups compared to a comparison group which acted as the control condition Participants: 330 Swedish university students with excessive alcohol consumption who had participated in a previous alcohol reduction app trial [14] Intervention: TeleCoach app consists of two main active components: tailored feedback and monitoring module with information; a skills training module with tools to reduce alcohol consumption Immediate access group: 93 participants given immediate access to the TeleCoach app Delayed-access group: 93 participants given access to the TeleCoach app after 6-weeks Control group: 144 assessment-only controls with excessive alcohol consumption from a previous trial [11] were used for comparison Follow-up: 6 and 12 weeks	During the follow-up period the odds for not having excessive weekly alcohol consumption in the immediate access group were almost twice as high as for controls (OR = 1.95, 95% CI = 1.36-2.80) At 12 weeks follow-up the immediate access group reported significantly fewer drinking days/week (P = 0.034) compared to controls No significant differences found in other measures (SD consumed/week, BD days/week, average eBAC/week and peak eBAC over a month) Availability: Unable to find app in any iTunes or Google Play stores
Minimise [17]	Young people seeking to reduce their alcohol consumption	Study design: Pilot RCT Participants: 45 participants aged 18-35 seeking to reduce their alcohol consumption recruited via social media and from ads placed at an Australian university campus Intervention group: 25 participants received the Minimise app, which is designed to deliver a range of protective behavioural strategies tailored to the user’s drinking goal (i.e., reduce consumption or drinking-related consequences), their momentary affective state (negative/positive), and their social, interpersonal context (who they are with). Control group: 20 participants received the InstantSurvey app (control app with only alcohol consumption-tracking functionality enabled). Follow-up period: 28 days	No significant reductions in frequency of risky drinking occasions (≥ 5 SD in a single setting) and alcohol-related harms (e.g. physically unwell, work/study consequence) from baseline to post-intervention in either group However, participants in the intervention group reported using protective behavioural strategies to control alcohol consumption over the past 2 weeks significantly more frequently than the controls (1.61 vs 1.07 times, P = 0.04), and to reduce alcohol-related harm when drinking significantly more frequently (1.47 vs 0.63 times, P = 0.01). Availability: Not available on any iTunes or Google Play as the authors have taken the app down to update it
D-ARIANNA Digital-Alcohol Risk Alertness Notifying Network for Adolescents and Young Adults [18]	Young people engaging in binge drinking	Study design: Natural, quasi-experimental, pre-post test design without a control group Participants: 590 participants aged 18-24 years recruited at pubs/clubs and music events Intervention: All participants were asked “did you binge drink in the past two weeks?” with a yes/no response, then were assisted to download the D-ARIANNA app onto their phones and were observed by facilitators to self-administer the app modules at least once. The app estimates current risk of BD by completion of questionnaires and gives a personalised risk estimate indicating how users’ risk factors contribute to the overall score Follow-up: Participants were phoned 14 days later and asked whether they had engaged in BD during this period. 83 of the 590 participants could not be reached	If the response from the 83 people lost to follow-up were assumed to be the same at the end of the study period as the start, then use of the app was associated with a statistically significant reduction in the proportion of participants reporting BD over the 2-week study period compared to baseline OR = 0.45 (95% CI, 0.37-0.55) Availability: Unable to find app in any iTunes or Google Play stores.
Drinks meter [19]	Young people who are current drinkers	Study design: Pilot RCT with two intervention and two control groups. Participants: 488 current drinkers aged 18 to 30 years recruited via social media, and posters/emails targeted at university students Drinks Meter iPhone/Android app group: 123 participants received the Drinks Meter app, which provides users with brief screening and advice for alcohol plus normative feedback, information on calories consumed and money spent OneTooMany web-based app group: 123 participants received a link to the OneTooMany website/web app, which presents users with a series of socially embarrassing scenarios that may occur when drinking and asks them to score themselves according to if/how recently they had been experienced Control group 1: 121 participants were asked to imagine they were exposed to information about alcohol misuse, without actually receiving any information Control group 2: 121 participants were asked for baseline and follow-up measures only Follow-up: 4-weeks	The study failed to recruit and obtain sufficient follow-up data to reach a prior estimated power for detecting a difference between groups (estimated required sample size was 800, number recruited = 488, with follow-up data on 402) Of the 402 participants sample with follow-up data there were no significant differences after 4-weeks between intervention and control groups on AUDIT-C scores, drinking harms measure via questionnaire or occasions of pre-loading (consuming alcohol at home before a night out) Availability: Drinks Meter app is available in the US, Canadian, UK, Irish, Australian and NZ stores iTunes and Google Play stores. One Too Many is can be accessed on a web browser - http://onetoomany.co/
Once Upon a High (VoltEgySzer in Hungarian) [20]	Young people aged 14-18 years	Study design: Cluster non-randomised controlled trial Participants: 386 students aged 14-18 years recruited from 2 vocational and 2 high schools in Hungary. Intervention group: Students from 1 vocational school and 1 high school instructed to download the Once Upon a High app, an interactive gamified drug prevention app that contains interactive comics/cartoons and games, and information on the effects of psychoactive substances and where to find list treatment and support in Hungary Control group: Students from 1 vocational school and 1 high school who did not download the app. Participants only completed the baseline and follow-up surveys Follow-up: Participants completed a baseline survey and follow-up survey 2-months later. 246 students completed the 2-month follow-up (140 or 36% lost to follow-up)	*Alcohol use outcome:* There was no significant difference in past month alcohol use between the intervention and control groups (P = 0.014) The only statistically significant outcome was a greater decrease in energy drink consumption after 2-months in the intervention group compared to control group Availability: VoltEgySzer app available in Hungarian in the US, Canadian, UK, Irish, Australian, NZ and Hungarian Google Play stores. Unable to find app in any iTunes stores

SD, standard drink; BD, binge drinking; IRR, incidence rate ratio; AUD, Alcohol Use Disorder; AUDIT, Alcohol Use Disorders Identification Test; MI, motivational interviewing; eBAC, estimated blood alcohol content; BrAC, breath alcohol calculator; RCT, randomized control trial

### Apps for young people

The available evidence for the efficacy of alcohol reduction apps among youth is inconclusive, with results from these evaluations not showing a clear benefit in reducing alcohol consumption compared to control groups [14–21]. Randomised control trials (RCTs) were conducted for five of the seven apps [14–17, 19, 21], with only two of these finding any significant reductions in alcohol consumption outcomes in intervention compared to control groups [16, 21]. Of the other two app studies, one was a quasi-experimental, pre-post test design without a control group and found a statistically significant reduction in the proportion of participants reporting binge drinking after the two-week study period compared to baseline (OR = 0.45, 95% CI = 0.37–0.55) [18]. The other, a cluster non-randomized controlled trial, found no statistically significant difference in past month alcohol use between the intervention and control groups (P = 0.014) [20].

### Apps for adult populations

Of the 12 apps designed for use in adult populations that have been evaluated, four are aimed at individuals in the general population who want to reduce their alcohol intake [22, 23, 26, 30], one at working adults experiencing psychological distress [31], and seven at individuals with an alcohol use disorder (AUD) [24, 25, 27–29, 32, 33]. The results from these evaluations are more promising than the apps designed for youth, but results are still mixed. Seven out of 12 studies found significant reductions in alcohol consumption measures, four found no significant reductions and one found a significant increase in alcohol consumption in the intervention group. However, few of these trials were randomized controlled trials with sufficient sample sizes.

Of the four apps aimed at individuals in the general population, two RCTs have been conducted: one found significantly larger reductions in standard drinks consumed per week among participants in one of the app intervention groups compared to controls who were offered the same app with ‘active’ components removed (P = 0.03) [23]; one did not find a significant difference in standard drinks consumed per week between participants who were offered access to an app and controls who did not receive an intervention (P = 0.17) [22]. Of the other two trials without control groups, one found a significant reduction in Alcohol Use Disorders Identification Test-C (AUDIT-C) scores (P < 0.001) at 3-months follow-up [30], and the other found a significant reduction in alcohol consumption only among ‘engaged app users’ [26].

Of the seven apps for use among individuals with AUD there have been three RCTs. One found a significant reduction in ‘risky drinking days’ among participants using an app compared to controls given only treatment as usual (P = 0.003) [25], while the other two trials found no significant differences in any alcohol consumption outcomes between the intervention and control groups [29, 32]. The other four trials in this population had mixed results, with three finding significant reductions in alcohol consumption measures and one finding no significant changes.

### Availability of apps in commercial app stores

Of the 19 alcohol reduction apps that have been evaluated [14–34] as far as we can ascertain only eight of these are currently publicly available in commercial app stores [15, 19, 20, 22–24, 26, 30, 34]. Of these eight apps, only four were demonstrated in the literature to lead to a reduction in alcohol consumption measures [23, 24, 26, 30, 34]. Two of these apps are exclusively available in the United Kingdom (UK) iTunes stores [23, 34], one is available in the Australian, New Zealand, United States, UK, Irish and Canadian iTunes stores [24], and the other can be accessed from the aforementioned countries’ iTunes and Google Play stores but comes at a cost after a 21-day trial period (except for Australian users) [30].

## Discussion

Despite the limited number of apps for managing alcohol consumption that have been evaluated in the literature, and the mixed evidence for their efficacy, there are currently hundreds of alcohol-reduction apps publicly available in the top commercial apps stores iTunes and Google Play. The situation is similar for other health issues, such as increasing physical activity [35]. There are hundreds of apps for increasing physical activity, however few are founded in an evidence-base [35].

### Commercial app store content analyses

Health researchers have begun to conduct content analyses of commercial app stores in order to identify the highest quality publicly available apps in these stores that could potentially be recommended to health professionals and consumers (e.g. [36–38]). These types of analyses involve conducting systematic searches of commercial apps stores to identify publicly available apps purporting to target the issue of interest. Researchers then download the apps and assess them on various quality measures. While this approach is unable to generate evidence regarding the effectiveness of apps, it is a potentially useful method to identify apps that adhere most closely to current evidence-based guidelines and are of the highest technical quality.

There have been several content analyses that have reviewed publicly available apps for managing alcohol consumption [39–44], however most do not identify the apps they found to be the highest quality during their analysis specifically by name meaning a reader could not take advantage of these reviews to locate and use the apps identified as the best. One content analysis that does identify the highest quality apps by name is a 2019 study by Tofighi et al., who reviewed the iTunes and Google Play stores for free or low-cost apps claiming to target alcohol or other substance use and evaluated the apps’ functionality, aesthetics, and quality of information of using the validated Mobile App Rating Scale (MARS) [44]. The analysis identified the highest rated apps on the scale and provided descriptions and background information on each app [44]. Content analyses such as this could be useful for health professionals and consumers to narrow down the highest quality apps from the hundreds available, and supplement the lack of evidence in this area.

While evidence for the efficacy of apps to reduce alcohol consumption is still in its infancy, and initial results appear to be mixed, established evidence on digital health interventions more broadly does appear to show a benefit in reducing alcohol consumption over those receiving no intervention [10]. However, with the paucity of rigorously evaluated apps clinicians could understandably feel hesitant in recommending them to patients. This issue is common for all health-related smartphone applications, with some healthcare professionals and consumers calling for app regulation or certification to be put in place to ensure quality [45]. In lieu of regulation or greater evidence, several groups have developed checklists that aim to assist in assessing the quality of health apps [46, 47]. The UK Royal College of Physicians (RCP) Health Informatics Unit has produced a checklist for clinicians to assess the structure, functions, impact and overall quality of health apps [48]. The RCP checklist is designed to help clinicians to feel more confident about recommending health apps to patients [48].

## Conclusion

The evidence for alcohol reduction apps is promising but inconclusive. Few apps that have been evaluated in the scientific literature are currently available for download in commercial app stores. In the absence of more scientifically evaluated apps for reducing alcohol consumption, well-designed content analyses of commercial app stores could potentially assist in identifying the highest quality apps that are publicly available.

## Data Availability

Not applicable.

## Abbreviations

AUD: Alcohol Use Disorder
AUDIT: Alcohol Use Disorders Identification Test
RCT: Randomised control trials
SD: Standard drink
BD: Binge drinking
IRR: Incidence rate ratio
MI: Motivational interviewing
eBAC: Estimated blood alcohol content
BrAC: Breath alcohol calculator
